# CD4 memory has a hierarchical structure created by requirements for infection-derived signals at an effector checkpoint

**DOI:** 10.3389/fimmu.2023.1306433

**Published:** 2023-12-12

**Authors:** Susan L. Swain

**Affiliations:** Department of Pathology, University of Massachusetts Chan Medical School, Worcester, MA, United States

**Keywords:** immune memory, pathogen, antigen, CD4, effector

## Abstract

Our recent studies reveal that the persistence, location, and amount of both antigen and signals that induce pathogen recognition responses determine the number of CD4 memory cells, the subsets that develop, their location, and hence their protective efficacy. Non-replicating vaccines provide antigen that is short-lived and generate low levels of only some memory subsets that are mostly restricted to secondary lymphoid tissue. In contrast, exposure to long-lived replicating viruses and bacteria provides high levels of diverse antigens in sites of infection and induces strong pathogen recognition signals for extended periods of time, resulting in much higher levels of memory cells of diverse subsets in both lymphoid and nonlymphoid sites. These include memory subsets with highly potent functions such as T follicular helpers and cytotoxic CD4 effectors at sites of infection, where they can most effectively combat the pathogen early after re-infection. These effectors also do not develop without antigen and pathogen recognition signals at the effector stage, and both subsets must receive these signals in the tissue sites where they will become resident. We postulate that this leads to a hierarchical structure of memory, with the strongest memory induced only by replicating pathogens. This paradigm suggests a likely roadmap for markedly improving vaccine design.

## 1 Introduction

Our lab has identified the mechanisms that generate long-lasting, potent CD4 memory responses that can protect us from the most dangerous pathogens. We have been humbled by how evolution has selected so many complex subsets of T-cell memory with diverse protective mechanisms and correspondingly complex pathways regulating their development (1). Here we discuss recent studies that bring into focus the mechanisms that drive the development of heterogenous subsets of memory at the effector phase of the CD4 response and what implications the signals required have for designing vaccines.

## 2 Background and recent studies

### 2.1 Generation of effector subsets from naïve CD4 T cells

Early studies focused on identifying the signals that are responsible for initiating the generation of effector cells from naïve CD4 T cells. Initiation of the naïve CD4 response requires cognate interactions between naive CD4 T cells with specialized antigen-presenting cells (APC) and is mediated by T-cell receptor recognition of peptide antigen bound to MHC-II and binding of costimulatory molecules on APC and T cells (2). The APC and CD4 T cells produce cytokines (IL-4, IL-6, IL-1, IL-2, and IFN-γ), which support the development of multiple CD4 effector subsets (e.g., Th0, Th1, Th2), themselves polarized to make distinct patterns of cytokines (3, 4). Viral infections induce mostly Th1 effectors, which participate in viral control, that once antigen is cleared, contract with a cohort differentiating to memory. These memory cells persist and are powerful mediators of protection (5, 6). They are the precursors of secondary effectors that clear viruses and provide future protection (6, 7).

CD4 T-cell memory responses to influenza A virus (IAV) infection are multifunctional, with distinct subsets mediating viral clearance, helping CD8 and B-cell responses, and producing cytokines that participate in viral control (4–7). Memory cells against influenza are found in secondary lymphoid organs (SLO), mediastinal draining lymph nodes, the spleen, and in infected tissues, including the lung (4–6). The CD4 memory subsets synergize with IAV-specific CD8 T and B cells (1) to provide strong, long-lasting protection against re-challenge with lethal doses of IAV against multiple IAV strains (7).

The process of generation of the initial CD4 effector subset (Th0, Th1, Th2) is well-defined at both cellular and molecular levels (3, 4). It can be readily modeled *in vitro* and requires only the initial signals from the recognition of APC and cytokines from CD4 and APC (2–4). However, more specialized subsets of CD4 T cells, such as T follicular helpers (T_FH_) (8), require further signals and have only been uncovered in more recent studies. Shane Crotty has defined the transcription factors needed for T_FH_ differentiation, especially Bcl6 (8). We defined key mechanisms that generate functionally distinct effector CD4 subsets from the mostly Th1 effectors present 6 days after IAV infection. We compared the generation of cytotoxic CD4 T cells (ThCTL) (9), which are found in infected lungs, and clear MHC-II^+^ virus-infected cells and T_FH_ that interact with B cells and drive differentiation in germinal centers to high-affinity antibody (Ab)-secreting cells (10). In contrast to initial effector subsets, which develop by the peak of the effector response 5–7 days postinfection (dpi) (6), T_FH_ and cytotoxic ThCTL develop only 2–3 days later. Their development requires another round of multiple signals during the effector stage, from recognition of antigens and pathogens to becoming memory cells (10, 11). Generation of both requires these signals in the local tissues, where these CD4 effectors become tissue resident and subsequently give rise to memory cells (10, 11). We propose these requirements for high levels of antigen and pathogen-derived products and create an additional checkpoint at the effector stage, which determines their fate and the make-up of late effector and memory responses (10–12).

### 2.2 CD4 effector cells are susceptible to programmed and activation-induced cell death

Often, vaccines that are composed of inert antigens induce short-lived effector CD4 responses with narrow specificity and modest development of immune memory against viruses (13). In contrast, previous infections with the same viruses can induce life-long protective immunity (13). We reasoned that this may be because most CD4 effectors generated by nonviable vaccines undergo wholesale default programmed cell death when survival cytokines are withdrawn (14, 15). This limits memory generation. We found that in *in vitro* models of CD4 effector generation from naïve CD4 T cells with antigen and activated APC, CD4 effectors undergo default programmed cell death, but they could be rescued by the addition of IL-2 and TGF-β (16). Recently, we followed CD4 effector fate *in vivo* after influenza infection and analyzed the signals from infection responsible for rescuing effectors from default apoptosis so that they could become memory cells (17). The infection-induced CD4 effectors required signals from autocrine IL-2 to effectively differentiate into long-lived CD4 memory (18). CD4 memory generation was drastically reduced when CD4 effectors could not make IL-2, but could be restored by IL-2-anti-IL-2 complex (IL-2C) treatment at 5–7 days postinfection (18). Without IL-2C, the IL-2 had to be autocrine and made during the cognate interaction of CD4 effectors with APC. The *in vivo*-generated effectors that develop in wild-type mice in response to influenza make 100- to 1,000-fold more memory than effectors from IL-2-deficient mice (18).

### 2.3 An effector checkpoint dictates CD4 memory development

#### 2.3.1 Role of antigen recognition

To further analyze what drives memory generation from effectors, we developed a sequential transfer model in which 6 dpi CD4 effectors were generated from naïve CD4 T-cell receptor (TCR) transgenic cells in first hosts infected with IAV. At 6 dpi, they were isolated and transferred to a second host in which we manipulated the availability of APC and IAV infection and monitored donor effector differentiation into memory. In the second host, recovery of almost all donor-derived memory required infection with IAV expressing the peptide seen by the TCR transgenic effector CD4 T cells, but could not be induced by IAV lacking that epitope (19). However, when we introduced APC activated with pathogen-recognition (PR) signals and pulsed with the peptide antigen, memory was fully restored (19). Memory generation depended on CD4 effector (autocrine) IL-2 production for 3 days beginning as effectors were generated (20), defining a checkpoint for memory. This reduced the programmed cell death of effectors and restored robust donor CD4 memory that protected the uninfected hosts from re-challenge (18–20). We concluded that, in order to survive and become memory cells, CD4 effectors generated by infection must again receive signals from cognate recognition and costimulation, resulting in autocrine IL-2, to which they respond and become memory.

We analyzed the impact of the avidity of TCR:peptide/MHC-II interaction at the checkpoint for memory generation. We developed a new CD4 TCR transgenic mouse that recognizes a dominant influenza nucleoprotein (NP) determinant and made a series of NP peptides with a broad range of avidities for the TCR (21). We pulsed activated APC with high concentrations of each peptide to evaluate the impact of avidity on NP-specific CD4 T cells. We found that higher TCR avidity for peptide-MHC-II proportionally increased memory generation (21). Moreover, the impact of avidity on responding effectors was already apparent in their survival after only 3 dpi and was also proportional to the amount of IL-2 produced by the responding effectors, which was strictly driven by avidity and peptide dose. This supports the concept that autocrine IL-2 acts directly to support effector survival and explains how the strength of TCR signals determines the extent of CD4 memory generation (21).

#### 2.3.2 Role of pathogen recognition pathways via activation of APC

**Figure 1:**
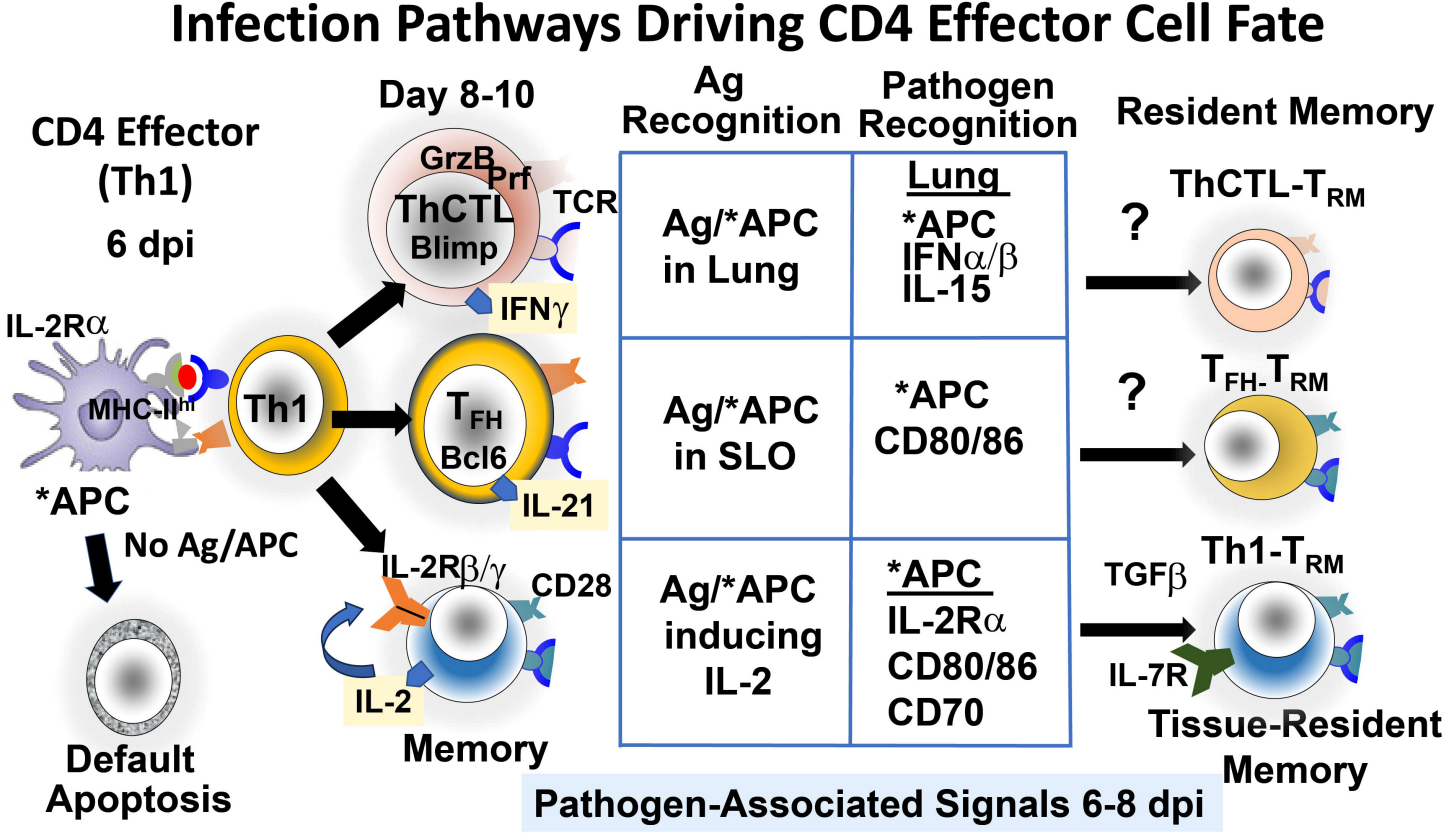
Pathways that drive CD4 effector cell fate at the effector checkpoint. Signals from continuing infection in the lung drive Th1 effectors to further differentiate to more specialized T_FH_ and ThCTL and likely to memory and tissue-resident memory (T_RM_) subsets. Signals include abundant viral antigen (Ag) presented by antigen-presenting cells (APC), activated by pathogen recognition (PR) pathways. (*APC) and by various cytokines secreted by other cells activated by PR pathways.

When activated by PR pathways, APC expresses high levels of surface MHC-II that enhance antigen presentation and multiple costimulatory ligands, including CD80 and CD86, that bind to ligands on the CD4 T cells so they produce IL-6, which is needed for the strongest naïve CD4 responses (22) (**Figure 1**). Replicating viruses such as influenza provide high levels of both the peptide antigens (20, 23) and the mediators induced by pathogen-derived signals (11). This is in contrast to most non-replicating vaccines, such as inactivated IAV, that supply antigens and associated adjuvants that rapidly decrease with time (23). Thus, many vaccines likely provide inadequate signals for CD4 effector progression to memory at the checkpoint.

We found that IAV infection induces expression of the IL-2 receptor β and γ chains on CD4 effectors, but many effectors did not express IL-2Rα (22). However, *in vitro* TLR agonist-activated APC and *in vivo* APC 6 dpi in infected mice did upregulate IL-2Rα, and APC that could not express IL-2Rα supported less memory generation than wild-type APC (21). These results suggest that IL-2 is trans-presented to the effectors by activated APC. Trans-presentation of IL-15 is a common mechanism (24), but IL-2 trans-presentation has rarely been identified (25). We suggest that the APC trans-presentation of IL-2 to the responding effectors amplifies the effective concentration of IL-2 during APC : CD4 effector cognate interaction (21). If so, the activation of IL-2Rα on APC by PR pathways is another mechanism by which infection-stimulated PR pathways contribute to CD4 memory generation. The known requirements we identified for the generation of CD4 memory from effectors are shown in **Figure 1** (Memory).

### 2.4 Checkpoint signals regulate the generation of highly differentiated TFH and ThCTL effectors

IAV infection generates a population of CD4 effectors, which are mostly Th1 cytokine-polarized cells with a few Th17, and this population gives rise 2–3 days later to T_FH_ and ThCTL (10, 11). These more differentiated effectors play critical roles in viral clearance and protection: T_FH_ support the germinal center response and selection of high-affinity B cells, which provide optimum B-cell Ab responses (26), and ThCTL kill infected cells expressing MHC-II (9), thus counteracting viral evasion mechanisms that downregulate MHC-I. We postulate that each effector then gives rise to distinct memory CD4 cells of related function, including T_RM_ memory, in tissue sites where the effectors reside (10, 11). We asked if the development of T_FH_ and ThCTL effectors from the 6 dpi Th1 effectors also requires antigen recognition and/or other signals from infection. When 6 dpi effectors were transferred to second hosts without antigen or infection, few T_FH_ or ThCTL developed. Even the transfer of peptide-pulsed APC could not restore T_FH_ (10) or ThCTL (11) generation, though they restored memory production (19). Thus, virus infection, in addition to antigen recognition, was required for both these subsets (**Figure 1**). T_FH_ and ThCTL development is programmed by gene pathways on the opposing Bcl6-Blimp1 axes (9, 26). CD28 costimulation is required during this interaction for T_FH_ development but not for ThCTL generation, and only ThCTL is required for infection-induced 1L-15 (11), supporting the dichotomy between T_FH_ and ThCTL effector stage differentiation.

Both T_FH_ and ThCTL effectors express a gene program associated with tissue residence (loss of S1PR1, Klf2, CCR7) (10, 11, 26). Since T_FH_ are found almost exclusively in secondary lymphoid sites vs. ThCTL in the lung (the site of IAV infection), we asked if local antigen presentation is needed. We restricted presentation to the lung and draining lymph node (dLN) by introducing the APC intranasally and restricted presentation to the spleen with intrasplenic injection (10, 11). The usual intravenous route resulted in presentation mostly in the spleen. T_FH_ developed in dLN with i.v. introduction and in the spleen with i.s. or i.v., while lung ThCTL in the lung required i.n. inoculation (10). We suggest that local antigen presentation contributes to inducing residency and drives the final differentiation to T_FH_ and ThCTL (10, 11). Additional PR-induced pathways independent of how infection impacts APC, are also required for the development of T_FH_ and ThCTL (IL-15 and IFN type I) (10, 11). Unlike memory responses, the development of T_FH_ and ThCTL effectors from earlier effectors does not require IL-2 (10, 11), distinguishing the pathways to memory (18) from those of more differentiated effectors. We are currently investigating if the tissue-resident effectors can become resident memory CD4 T cells (CD4 T_RM_).

Both ThCTL and T_FH_ exert potent effector functions that, while critical for protection, have also been implicated in autoimmune diseases and disease pathologies (27, 28). Thus, it is likely beneficial that their development at this late effector checkpoint is tightly regulated by multiple concurrent signals of pathogen infection: antigen presentation, costimulation, and other signals generated by infection, as these requirements ensure T_FH_ and ThCTL only develop when infection continues through the effector checkpoint, indicating a more potent immune response is needed to effectively mediate viral clearance and induce strong memory.

### 2.5 Hypothesis: the generation of distinct CD4 effectors and additional memory subsets by infection results in greater protection and a hierarchy of memory

**Figure 2:**
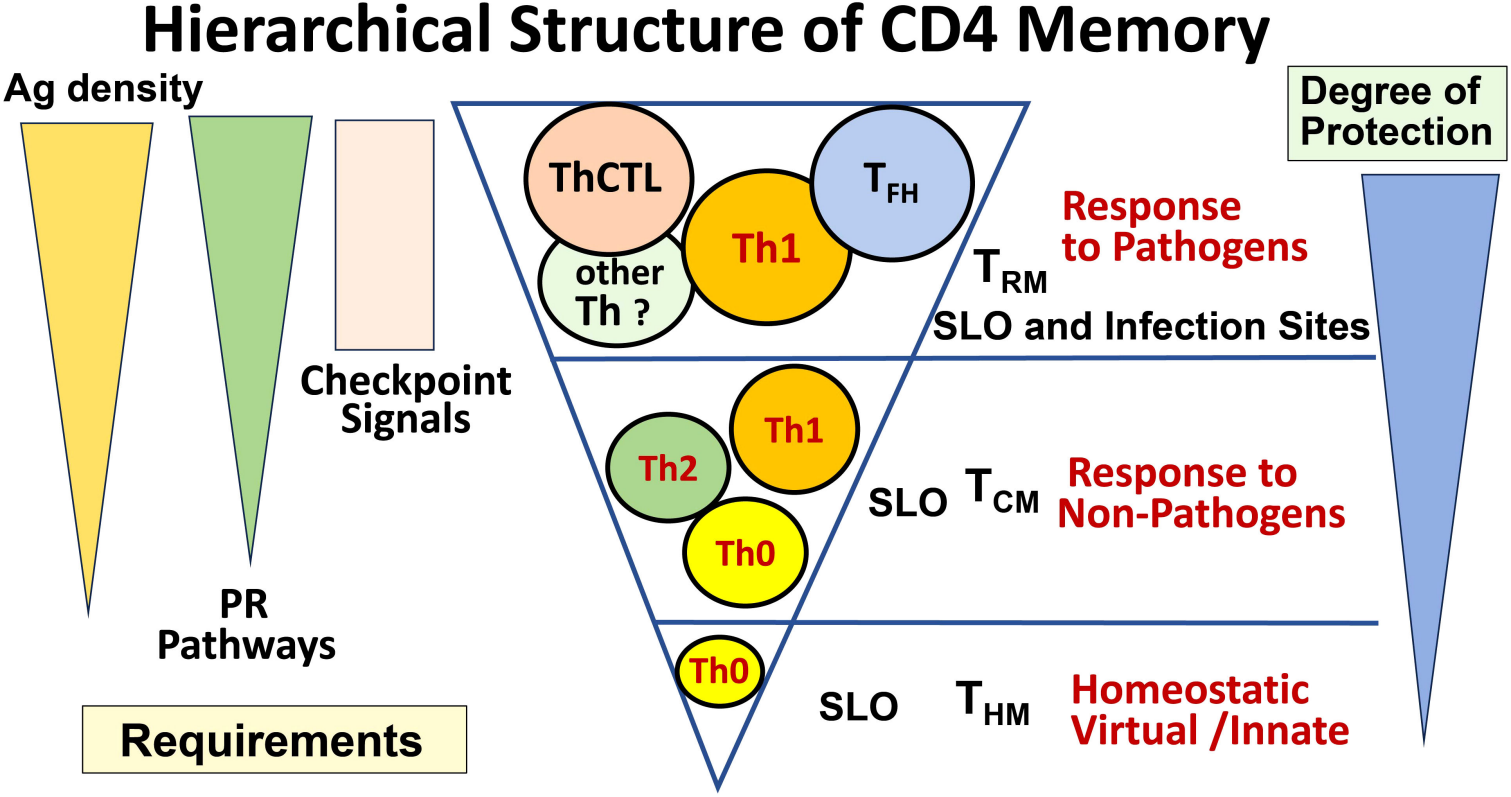
Hierarchical structure of CD4 memory. The signals driving the generation of CD4 effector and memory subsets result in a very different spectrum of memory subsets depending on the nature of the stimulus. When no foreign Ag is present, only a small number of homeostatic (aka innate, virtual) memory cells develop (T_HM_ bottom). When noninfectious Ag is present at sufficient levels, especially if accompanied by a source of PR signals, cytokine-polarized effectors are generated, and they can generate some central memory (T_CM_ middle). When a pathogen is present that persists into the effector phase, such that high levels of Ag and PR signals persist in infected tissues, high numbers of more specialized effectors (T_FH_, ThCTL) can be generated, and we postulate that these give rise to tissue-resident memory (T_RM_). We predict that the highest level of long-lasting protection requires these specialized effectors and memory from them.

CD4 T-cell immunity is important because T cells often target internal conserved proteins shared among heterosubtypic virus strains, presented by MHC-II, which is not readily downregulated by viral mechanisms, and thus they respond to emerging viral variants that frequently mutate their surface proteins that are targeted by Ab (8). The strict requirements at the CD4 effector stage described here to generate memory directly, and the T_FH_ and ThCTL effectors and memory they become, are readily supplied by replicating viruses but not by benign foreign or self-antigens. Infection drives more memory cells, memory cells with more potent functions, and in sites of potential re-infection, providing greater protection against replicating pathogens (**Figure 2**). Thus, we postulate the checkpoint requirements we have defined, create a striking hierarchy of CD4 memory. Those memory cells generated by homeostatic exposure to self-antigen (29) induce memory in the central sites (T_CM_) of the secondary lymphoid organs (SLO) that are Th1-like (30). Exposure to foreign, but nonreplicating, antigens, even if of high affinity and associated with some adjuvant, gives short-lasting signals and induces memory mostly directly from initial CD4 effectors (Th0, Th1, Th2, etc.) and mostly in central sites (T_CM_). In contrast, infection, producing high levels of antigen and PR recognition signals through the effector checkpoint, induces the greatest functional diversity, additional more potent CD4 memory subsets, and memory that is resident in the tissues of infection as well as SLO (**Figure 2**).

## 3 Discussion

The above paradigm for posteffector CD4 effector differentiation leads to a hierarchical scheme of CD4 memory with ascending functional diversity, tissue residence, and potency in clearing infection. They each correlate with multiple, synergizing signals they receive at the effector stage checkpoint: the level, avidity, and duration of antigen presentation, the level of activation of antigen-presenting cells, and the level of signals from other pathogen recognition pathways. This hierarchy should restrict the development of the most potent, and hence risky, CD4 immune memory to infection with the most dangerous pathogens.

### 3.1 Unanswered questions and limitations

The paradigm that CD4 effectors require re-exposure to high levels of Ag and pathogen recognition signals for memory and T_FH_ and ThCTL development has not yet been definitively verified in humans or nonhuman primate models. Also, long-lived humans accumulate a higher proportion of memory cells as they age, and thus more responses may be derived from CD4 memory and fewer from naïve CD4 T cells. However, our studies in mice suggest that memory CD4 T cells have equivalent requirements for these signals to make secondary memory cells (21). We do not know if other CD4 effector subsets may also require checkpoint signals. CD8 T-cell memory benefits from prolonged antigen exposure (31, 32), and CD8 T_RM_ requires recognition at the site of residency (33, 34, 35). Whether a comparable set of requirements applies to CD8 effectors and creates a comparable hierarchy is not established, but it seems likely, considering they follow a parallel scheme of development.

Our studies define only the role of Ag and PR signals at the checkpoint where effectors have formed and their proximal fate, whether apoptosis or further differentiation to T_FH_, ThCTL, or memory, is determined (**Figure 1**). In our transfer studies and in acute infections that are effectively cleared, both signals soon disappear, although Ag may persist in some specialized APCs. In situations of chronic infections or continuing exposure to Ag, exhaustion of CD4 effectors can occur. An advantage of vaccine strategies is that they can be designed to provide signals through the effector phase but not much longer thereafter.

### 3.2 Implications for vaccine designs

We suggest a major weakness of many current non-replicating vaccines is their failure to provide the prolonged antigen and pathogen signals needed to support high levels of CD4 memory and T_FH_ and ThCTL subsets (10, 11, 17, 19, 23). Indeed, inactivated influenza presents CD4 antigens for only a few days, producing mostly Th0 and Th1 effectors in the SLO with few T_FH_, limited generation of B-cell Ab. The addition of live, attenuated influenza at the effector peak enhances T_FH_, effectors in the lung, and Ab production (23). Protein vaccines present only a fraction of viral epitopes. CD4 T cells’ memory generation depends on high affinity and peptide density on APC during the cognate interaction of CD4 effectors at the checkpoint, which determines the level of IL-2 and hence the extent of memory generation. However, functional avidity depends on peptide interaction with MHC-II and that will vary with individual alleles. This argues that the extent of CD4 T-cell memory generation in a human population will increase with the breadth of antigens included in the vaccine. Inducing CD4 memory to more epitopes will also provide less chance viral variants have of escaping CD4 immunity.

We need vaccines to generate strong multifunctional CD4 memory against potentially deadly pathogens such as influenza and SARS-CoV2 that rapidly mutate and evade Ab-mediated protection. Current vaccine immunity is short-lived, and both these infections require frequently updated vaccines to the new target selected mutants.

We predict that the pathways we have defined in influenza will be found to be equally applicable to many other viral infections. Thus, we suggest that providing missing signals to current vaccine platforms could make them much more effective. In mouse models, we have provided: *in vivo* peptide antigen on TLR-activated APC (18, 19, 23), IL-2:anti-IL-2 Ab complexes (18), infection surrogates (10, 19, 23), and others that have supplied PR signals in humans (36) and all enhance CD4 memory and protection. Thus, there is support for the concept that providing the missing signals, at the effector stage, to a basic vaccine platform should improve CD4 immunity.

## Data availability statement

The original contributions presented in the study are included in the article/supplementary material. Further inquiries can be directed to the corresponding author.

## Ethics statement

The animal study was approved by Institutional Animal Care and Use Committee University of Massachusetts Chan Medical School. The study was conducted in accordance with the local legislation and institutional requirements.

## Author contributions

SS: Conceptualization, Funding acquisition, Project administration, Supervision, Writing – original draft.
